# 104. Evaluation of Vaccination Rates and Factors associated with Vaccine Uptake among People Living with HIV in Detroit, MI

**DOI:** 10.1093/ofid/ofac492.182

**Published:** 2022-12-15

**Authors:** Navina K Birk, Indira Brar, Lea M Monday, Tarandeep Singh, Medha R Cherabuddi, Marwa Hojeij, Brandon Ho, Anne Chen, George J Alangaden

**Affiliations:** Henry Ford Hospital, Detroit, Michigan; Henry Ford Hospital, Detroit, Michigan; Wayne state University School of Medicine, Detroit, Michigan; Henry Ford Hospital, Detroit, Michigan; Henry Ford Hospital, Detroit, Michigan; Henry Ford Hospital, Detroit, Michigan; Henry Ford Hospital, Detroit, Michigan; Henry Ford Hospital, Detroit, Michigan; Henry Ford Health, Detroit, Michigan

## Abstract

**Background:**

Determination of vaccination rates for people living with HIV (PLWH) and factors that affect adherence to vaccination is important to ensure these vulnerable patients are optimally protected against vaccine-preventable diseases. We analyzed the rates of vaccination and associated factors in PLWH receiving care in the Henry Ford Health Infectious Diseases (HFH ID) Clinic in Detroit, MI.

**Methods:**

We implemented a retrospective, observational study. Inclusion criteria were all PLWH who had at minimum two clinic visits at HFH ID clinic within 12 months from 2015-2021.

Charts were reviewed for demographic data. We analyzed the rates of all eligible vaccines including the hepatitis A and B, HPV, influenza, pneumococcal, tetanus, zoster, and COVID-19 vaccines.

**Results:**

A total of 661 met the inclusion criteria. Average age of the patients was 50 years. 78.6% were male, 74.3% black, and 57.6% patients were from Detroit. On average, patients had 1 clinic visit in the past year at HFH ID Clinic.

Rates of influenza, pneumococcal, and tetanus vaccinations were above 90%. Rates of hepatitis A and B vaccinations were above 80%. Rates of zoster and HPV vaccinations were above 50%. COVID-19 vaccination had the lowest rate at 42.1%. Patients who had received all recommended vaccines were more likely to be male, have a HFH PCP, men who have sex with men (MSM), younger, more HFH ID clinic visits, and have a higher CD4 count on entry into care. Factors associated with increased vaccine uptake include having a HFH primary care physician (PCP), more HFH ID clinic visits, and a CD4 count above 200 on entry. Having 2 clinic visits in the previous year was associated with a higher likelihood of vaccine adherence [OR 5.85 (95% CI 0.360 – 0.723)].

**Figure:**
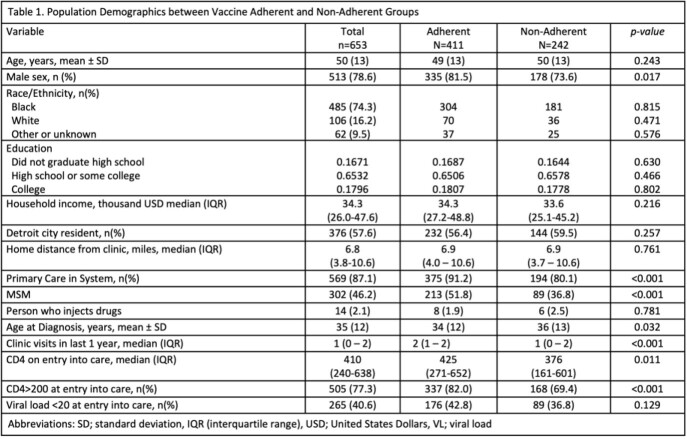

**Figure:**
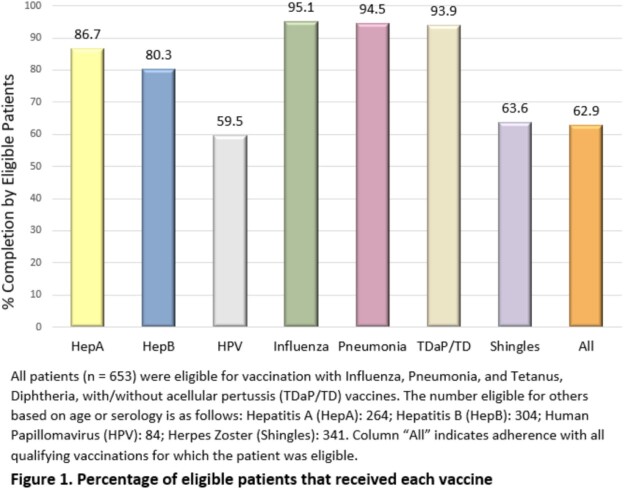

**Figure:**
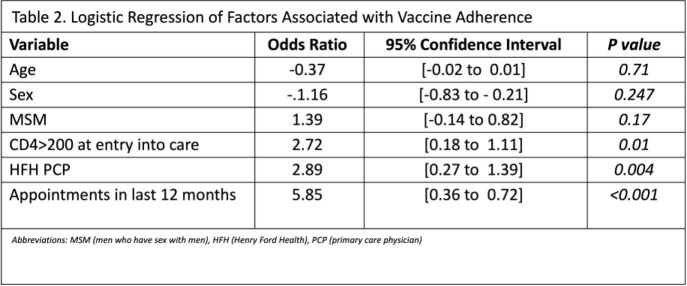

**Conclusion:**

Our study shows that even in this highly vulnerable, vaccine-hesitant population, programs that integrate vaccines and promote adherence to clinic care into the routine care of PLWH results in high rates of vaccine uptake.

**Disclosures:**

**Indira Brar, MD**, Gilead: Grant/Research Support|Gilead: speakers bureau|Janssen: Grant/Research Support|Janssen: speakers bureau.

